# A daily Azores–Iceland North Atlantic Oscillation index back to 1850

**DOI:** 10.1002/gdj3.23

**Published:** 2015-03-27

**Authors:** Thomas Cropper, Edward Hanna, Maria Antónia Valente, Trausti Jónsson

**Affiliations:** ^1^ Department of GeographyUniversity of SheffieldSheffieldUK; ^2^ Instituto Dom LuizUniversity of LisbonLisbonPortugal; ^3^ Icelandic Meteorological OfficeReykjavikIceland

**Keywords:** atmospheric science, climate, weather, North Atlantic Oscillation, daily

## Abstract

We present the construction of a continuous, daily (09:00 UTC), station‐based (Azores–Iceland) North Atlantic Oscillation (NAO) Index back to 1871 which is extended back to 1850 with additional daily mean data. The constructed index more than doubles the length of previously existing, widely available, daily NAO time series. The index is created using entirely observational sea‐level pressure (SLP) data from Iceland and 73.5% of observational SLP data from the Azores – the remainder being filled in via reanalysis (Twentieth Century Reanalysis Project and European Mean Sea Level Pressure) SLP data. Icelandic data are taken from the Southwest Iceland pressure series. We construct and document a new Ponta Delgada SLP time series based on recently digitized and newly available data that extend back to 1872. The Ponta Delgada time series is created by splicing together several fractured records (from Ponta Delgada, Lajes, and Santa Maria) and filling in the major gaps (pre‐1872, 1888–1905, and 1940–1941) and occasional days (145) with reanalysis data. Further homogeneity corrections are applied to the Azores record, and the daily (09:00 UTC) NAO index is then calculated. The resulting index, with its extended temporal length and daily resolution, is the first reconstruction of daily NAO back into the 19th Century and therefore is useful for researchers across multiple disciplines.

## Dataset

Identifier: doi: 10.5281/zenodo.9979

Creator: Cropper, L., Hanna, E., Valente, M. A., and Jónsson, T.

Title: Daily resolution Azores–Iceland station‐based North Atlantic Oscillation Index

Publication Year: 2015

## Introduction

The North Atlantic Oscillation (NAO) represents the principal mode of annual variability across much of the Atlantic sector of the Northern Hemisphere (Visbeck *et al*., 2001; Osborn, 2011). The NAO is traditionally defined as the difference in normalized sea‐level pressure (SLP) anomalies between a southern node, located in continental Iberia or the Azores, and a northern node, usually Southwest Iceland [(Hurrell, 1995; Jones *et al*., 1997), hereafter, the ‘station‐based’ method]. Alternatively, the NAO can be calculated from gridded climate datasets using empirical orthogonal analysis (EOF) or similar methods [(Thompson and Wallace, 1998; Folland *et al*., 2009), hereafter, the PC‐based method]. The advantage of the station‐based methodology is the extension back to the mid‐19th Century and a continuous temporal record from each node, allowing a consistent methodology for deriving the NAO. The shortcomings of the station‐based NAO are as follows: (1) the fixed spatial location of the weather stations and (2) noise due to transient and local meteorological events and, as discussed below, inhomogeneity of the southern station pressure series. The PC‐based NAO better captures the annual migration of the centres of action of the NAO dipole, which is particularly important during the boreal high‐summer months [July and August (Folland *et al*., 2009)], when the pattern typically reverts to a ‘Greenland‐British Isles seesaw’, instead of the usual ‘Azores‐Iceland’ pattern. The PC‐based indices are limited by the accuracy of the reanalysis products from which they are derived, the nonstationarity of the EOF pattern (Wang *et al*., 2014) and usually having to repeat the analysis every time an update is required which changes the previous historical values (Climate Data Guide, 2014: https://climatedataguide.ucar.edu/climate-data/hurrellnorthatlantic-oscillation-nao-index-pc-based).

The station‐based NAO almost always use the Southwest Iceland SLP time series as the northern node, which is a well‐documented daily SLP record extending back to 1823 (Jónsson and Gardarsson, 2001; Jónsson and Miles, 2001; Jónsson and Hanna, 2007). Three commonly used southern station nodes are Ponta Delgada (Azores, Portugal), Gibraltar (British Overseas Territory), and Lisbon (Portugal). The two continental locations are generally accepted as ideally located for representing the winter NAO (DJF), adequate for spring and autumn (MAM and SON, respectively), and unsuitable for summer (Hurrell and Van Loon, 1997; Jones *et al*., 1997). Pozo‐Vázquez *et al*. (2000) emphasized the importance of using the Azores station as the southern node if using a monthly or seasonal station‐based NAO. Monthly SLP records from Gibraltar, Lisbon, and the Azores extend back to 1821, 1864, and 1865, respectively. Several studies have sought to extend the temporal length of the NAO further back in time by the use of proxy‐based reconstructions, but these are usually winter‐based and/or based on potentially nonstationary assumptions about the proxy–NAO relationship (Luterbacher *et al*., 1999; Cullen *et al*., 2001; Schöne *et al*., 2003; Lehner *et al*., 2012). A recent reconstruction of the monthly NAO back to 1692 using London and Paris as the northern and southern nodes highlights the value in using recently digitized historical data to reconstruct the NAO (Cornes *et al*., 2013).

Our focus is to increase the temporal resolution of the NAO index in the form of a continuous, daily ~09:00 UTC index extending back to 1871 (and 1850 with mean daily data). Our reason for this is twofold. Firstly, it is apparent that the index, particularly in December, is undergoing a significant change in its variability towards more extreme values (Hanna *et al*., 2015) and, in contrast to global climate model simulations (Folland *et al*., 2009; Belleflamme *et al*., 2012), there has been a recent negative trend in the summer NAO (Hanna *et al*., 2015). Secondly, the consistency in the publication of Azores SLP values since 2003 has become increasingly sporadic (Cropper and Hanna, 2014). Also, it has recently become apparent that the published monthly SLP values from Lisbon in several meteorological archives are inhomogeneous (Bethke and Valente, 2012), which may propagate as errors into the NAO (as the index is normalized, the errors will be small, but improvements should be made where it is possible to do so). Furthermore, use of a consistent observing time when possible should minimize the effect of diurnal pressure tides (Dai and Wang, 1999). Typically, the diurnal pressure cycle for the Azores and Iceland peaks at ~05‐6:00/17‐18:00 UTC (minimum) and 23‐00:00/11‐13:00 (maximum) – with a (max–min) diurnal SLP range of ~2.0 and ~0.7 hPa, respectively (data not shown). The only widely available, consistently updated daily NAO index is provided by the Climate Prediction Centre (CPC, http://www.cpc.ncep.noaa.gov/products/precip/CWlink/pna/nao.shtml), who construct a daily index using a PC‐based method (Barnston and Livezey, 1987), which extends back to 1950. Previously, several studies have made use of daily NAO indices (Jónsson and Miles, 2001; Blessing *et al*., 2005; Philipp *et al*., 2007; Folland *et al*., 2009; Woollings *et al*., 2010), displaying the usefulness of an enhanced temporal scale in analysing the predictability, persistence characteristics, and evolution of the NAO. As such, a quality‐controlled daily NAO index that can be easily updated should be of great value to researchers across multiple disciplines. This study presents the efforts made to acquire and homogenize daily data from the southern node of the NAO, across the Azores, and use of the newly created, continuous Azores SLP time series to construct a daily (≅09:00 UTC) NAO index back to 1871 which we further extended to 1850 by the use of daily mean SLP output from reanalysis.

## 1 Methodology

### 1.1 Time of observation

The following information regarding the time zone history of Iceland and the Azores was taken from the latest release of the IANA Time Zone Database (ftp://ftp.iana.org/tz/data/europe). Coordinated Universal Time (UTC) was introduced on 1 January 1972, which superseded Greenwich Mean Time (GMT) (established 1 November 1884) as the international standard time. Throughout the article, ‘*z*’ refers to local time and UTC is the primary time standard (GMT from 1884 to 1971 and UTC 1972 onwards). The time zone of Iceland is UT0 (i.e. the same as Greenwich, UK), but Iceland did not adopt the global time zone until January 1908, when GMT‐1 was adopted. As such, based on the longitude of Reykjavik (338.11E), the local time of Iceland pre‐1908 is approximately 90 min behind GMT (so 09:00 UTC for Iceland pre‐1908 is ~07:30z). Iceland invariably observed daylight saving time between March/April and the end of October during 1917–1919 and 1939–1967 (http://www.almanak.hi.is/klukkan.html) and from 1968 Iceland has been on UT0 with no summertime observed.

Portugal adopted GMT in 1912 and it is assumed, but not certain, that GMT was adhered to for meteorological observations across Portugal from 1912, but it may also not have been until 1947, when the Institute of Meteorology was formed. Based on the longitude of Ponta Delgada (334.32E), the pre‐1912 local times from the Azores are ~110 min behind GMT (so 09:00 UTC for Portugal pre‐1912 is ~07:10z). The Azores adopted GMT‐2 in January 1912 and changed from GMT‐2 to its current UTC‐1 around September 1983. Daylight saving was introduced in 1916, and varied in when/if it was applied throughout the year until late in the 19th Century. The Azores and Iceland stations that are discussed below document how changes in the observation time vary between 06:00z and 12:00z in the early parts of the records (essentially, 08:00–14:00 UTC), but this will introduce a minimal amount of bias into the time series given the small range of diurnal pressure tides.

### 1.2 Northern NAO node (Southwest Iceland)

**Figure 1:**
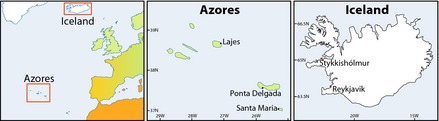
Location of the Azores and Iceland within the North Atlantic Basin and the location of the specific stations used in construction of the Azores and Iceland SLP time series.

The Southwest Iceland pressure series is a composite of fixed‐time, usually at 09:00 UTC daily readings from Stykkishólmur and Reykjavík since March 1822 (Figure 1). Jones *et al*. (1997) and Jónsson and Gardarsson (2001) describe the sources of the early Icelandic pressure data and Jónsson and Miles (2001) applied additional homogenization to the time series. The time of observation is usually around 07:00‐08:00z pre‐1920, and 07.30z/09:00 UTC post‐1920 (Jónsson and Hanna, 2007). The Southwest Iceland pressure series (Jónsson and Miles, 2001) is extended to December 2014 with data from the Icelandic Meteorological Office.

### 1.3 Southern NAO node (Ponta Delgada, Azores)

Monthly data from Ponta Delgada (1865–2000) are readily available [e.g. the ADVICE SLP archive (Jones *et al*., 1999)]; however, (sub‐)daily data have historically been difficult to acquire and this station has reported unreliably since 2003 (Cropper and Hanna, 2014). The ‘historical’ station which reported for ~140 years since 1865 has ceased operation and has been replaced by a site at Nordela Airport since 1973 (Table 1). Subdaily data from the Integrated Surface Data (ISD, http://www7.ncdc.noaa.gov/CDO/cdo) have been made recently available for several Azores stations, most noticeably from 1931 to 1961 for Ponta Delgada. In addition, data from Ponta Delgada extending back to December 1872 have been recently digitized (Table 1). Together, these new Ponta Delgada data have a long gap from 1888 to 1906 and a shorter one from December 1939 to 1941, but otherwise run relatively uninterrupted until 1961, which overlaps with alternative Azores pressure records (from Santa Maria and Lajes) and allows for a continuous time series to be constructed. It is advantageous that many of the early records have pressure data at fixed daily readings, usually within ±2 h of 09:00 UTC (Table 1), which is consistent with the Southwest Iceland data. The following three sections deal with: (1.3.1 and 1.3.2) the construction of two sections of the Ponta Delgada time series (1872–1961 and 1944–2013), 1.3.2 and (1.3.3) the filling in of gaps using 20th Century reanalysis (20CR) data (Compo *et al*., 2011); extension of the record back to 1850 with European mean sea‐level pressure (EMSLP) data (Ansell *et al*., 2006) and homogenisation of the constructed Ponta Delgada time series. The final result is a continuous, homogenized Azores daily SLP record extending back to 1850.

**Table 1:** The various data sources used to create the daily (~09:00 UTC) Ponta Delgada SLP time series from 1850 to 2013

ID	Location	Latitude	Longitude	Altitude (m)	Measurement time (UTC/z)	Temporal start	Temporal end	Source	Detail
Old PD	Ponta Delgada (São Miguel)	37.74N	334.32E	20	09:00z	December 1872	December 1887	IDL – SIGN	Instituto Dom Luiz, Valente *et al*. (2008)
Old PD	Ponta Delgada (São Miguel)	37.73N	334.33E	17	09:00z	January 1906	December 1914	IDL – ERACLIM	Valente *et al*. (2013)
Old PD	Ponta Delgada (São Miguel)	37.73N	334.33E	22	09:00z	January 1915	December 1922	IDL – ERACLIM	
Old PD	Ponta Delgada (São Miguel)	37.73N	334.33E	22	11:00z	January 1922	December 1935	IDL – ERACLIM	1 year gap during 1931
Old PD	Ponta Delgada (São Miguel)	37.73N	334.33E	22	06:00z	January 1942	December 1944	IDL – ERACLIM	
Old PD	Ponta Delgada (São Miguel)	37.73N	334.33E	22	07:00z	January 1945	December 1946	IDL – ERACLIM	
OPD(ISD)0600	Ponta Delgada (São Miguel)	37.73N*	334.33E*	22	06:00 UTC	January 1931	November 1939	ISD	Integrated Surface Data. *Assumed based on Old PD location
		37.73N**	334.33E**	22	06:00 UTC	April 1953	August 1961	ISD	**Pressure reading is only to nearest whole SLP
OPD(ISD)1200	Ponta Delgada (São Miguel)	37.73N*	334.33E*	22	12:00 UTC	January 1931	October 1939	ISD	*Assumed based on Old PD location
		37.73N**	334.33E**	22	12:00 UTC	January 1948	April 1953	ISD	**Pressure reading is only to nearest whole SLP
Modern PD	Ponta Delgada (São Miguel)	37.74N	334.3E	71	09:00 UTC	January 1973	Present	ISD	Nordela Airport
NPD(ISD)0600	Ponta Delgada (São Miguel)	37.74N	334.3E	71	06:00 UTC	January 1973	Present	ISD	Nordela Airport
NPD(ISD)1200	Ponta Delgada (São Miguel)	37.74N	334.3E	71	12:00 UTC	January 1973	Present	ISD	Nordela Airport
Lajes (SLP/STP)	Lajes air base (Terceira)	38.76N	332.91E	55	09:00 UTC	January 1947	Present	ISD	
Santa Maria	(Santa Maria)	36.97N	334.83E	100	09:00 UTC	August 1944/March 1966	Present	ISD	
20CR	Ponta Delgada (São Miguel)	38N	334E	Sea level	09:00 UTC	January 1871	December 2011	Compo *et al*. (2011)	09:00 UTC is average of 06:00 and 12:00 SLP data
EMSLP	Ponta Delgada (São Miguel)	35N	335E	Sea level	Daily average	January 1850	December 2003	Ansell *et al*. (2006)	SLP is a daily value, not 09:00 z

*/** indicates when metadata assumptions were made or when precision issues were present in the source data.

#### 1.3.1 Historical Azores SLP data (1872–1961)

Recently digitized Ponta Delgada (Azores) data (Table 1) run from 1872 to 1887, 1906 to 1930, 1932 to 1935, and 1942 to 1946. The 19th Century data for 1872–1887 were digitized through project SIGN (Valente *et al*., 2008) and the early 20th Century (1906–1946) by project ERA‐CLIM (Valente *et al*., 2013). During 1872–1887 and 1906–1921, pressure readings were taken at 09:00z (local time, equivalent to ~10:50 UTC). The location of the station slightly changes from an altitude of 20 m (37.74N, 334.32E) before 1888 to 17 m between 1906 and 1914 and to 22 m (37.73N, 334.33E) from 1915 onwards. During 1922–1930 and 1932–1935, pressure readings were taken at 11:00z. During 1942–1944 and 1945–1946 pressure readings were taken at 06:00z and 07:00z, respectively. All data were digitized as station pressure (STP) and converted to SLP after applying corrections for gravity, temperature, and altitude (observational temperature data are not yet available, so a set value of 289.15 K was used). After conversion to SLP, we treated the digitized Ponta Delgada data as a continuous series.

Ponta Delgada data from the ISD archive were provided directly as SLP. Daily readings at 06:00 UTC run from 1931 to 1939 and 1953 to 1961 and at 12:00 UTC from 1931 to 1939 and 1948 to 1953. When possible, we created a 09:00 time for each day by the average of the 06:00 UTC and 12:00 UTC reading. We spliced together a historical Ponta Delgada record by least squares linear regression of the ISD data against the digitized Azores data (based on the 1932–1935 overlap period), which created a historical Ponta Delgada time series from 1872 to 1961 without any large gaps (>1 year) between 1906 and 1939 and 1942 and 1961 (Table 2). Preference was given to 09:00/06:00 UTC ISD data when gap filling. The regression splicing served as a basic quality control for identifying and removing significant outliers (with regression coefficients being recalculated after outlier removal). Obviously incorrect SLP values (below/above 950/1050 hPa) were also removed.

#### 1.3.2 Modern Azores SLP data (1944–2013)

**Figure 2:**
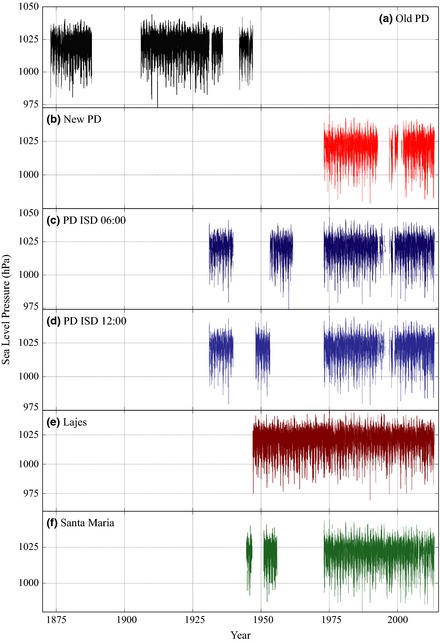
Daily time series of the available raw (unhomogenized) meteorological station SLP data from the Azores.

09:00 UTC data from the ISD Ponta Delgada station run uninterrupted from 1973 to 1992 and 2002 to 2014, with significant gaps present between 1992 and 1996 and 1999 and 2002. 06:00 UTC and 12:00 UTC data adequately cover the 1999–2002 gap and provide somewhat limited coverage between 1992 and 1995. This Ponta Delgada record was extended as before by regression against the 09:00/06:00/12:00 UTC ISD data (based on the long‐term 1973–2013 overlap period); however, this did not cover all of the gaps in the time series or extend the data back to a period where it could be spliced against the old Ponta Delgada record to form a continuous series. To do this, we use 09:00 UTC data from two nearby stations from Santa Maria (the island 80 km to the southwest of São Miguel) and Lajes Air Base (on Terceira, 170 km northwest), which report back to August 1944 and January 1947, respectively (Table 1). 09:00 UTC data from Santa Maria were provided directly as SLP values and run from August 1944 to September 1946, 1951–1955, and 1973 to present. 09:00 UTC readings from Lajes were incompletely provided as SLP, with the period March 1966–January 1973 only provided as STP (a significant period given the gaps in the Ponta Delgada records, Table 1). We corrected STP values to SLP as before (with use of 09:00 UTC temperature data when available or 289.15 K when a temperature reading is unavailable) and checked that our calculated SLP values were consistent with the given SLP values on days when both were available. Figure 2 displays the time series of all the individual Azores station data sources. The Santa Maria and Lajes records (using Santa Maria with priority due to its closer proximity) were regressed against the 1973–2013 Ponta Delgada record, which extended the modern 09:00 UTC daily Ponta Delgada pressure record back to August 1944 (with only ten missing days up to present).

#### 1.3.3 Completing the Azores SLP record

**Figure 3:**
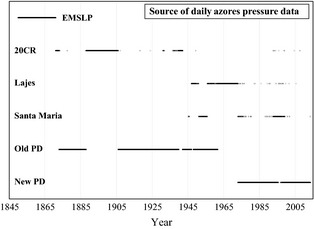
The source of daily pressure data used in the creation of the finalized Azores/Ponta Delgada time series.

The fully extended ‘historical’ (1872–1961) Ponta Delgada record was then regressed against the ‘modern’ (1944–2013) record to create a long‐term Ponta Delgada time series (December 1872–October 2013). The main gaps in this initial record were 1888–1905 and December 1939–1941, with only 135 single days missing outside these periods. To create a fully complete, continuous record, we used the grid box that overrides São Miguel from the 20CR dataset (Table 1). The 20CR has a 4× daily resolution for SLP, so we used the average of the 06:00 UTC and 12:00 UTC daily values as a proxy for 09:00 UTC, which was then regressed against the long‐term Ponta Delgada series. The linear relationship between the 20CR and observational data (Table 2) is strong (*r* ^2^ = 0.931). The 20CR‐filled Ponta Delgada time series runs continuously from 1 January 1871 to December 2014. An extension of the PD record back to 1850 is calculated using additional daily data from the EMSLP project; however, the EMSLP readings are daily averages, which differ from our typical ~09:00 UTC readings (the number of daily observations that create the daily average does vary temporarily and is a potential source of bias). The EMSLP output is on a 5 × 5° grid and during 1850–1880, 85–90% of the daily grid cells have missing data, so a large fraction of the SLP values are constructed based on the reduced space optimal interpolation procedure used (Ansell *et al*., 2006). As such, one might expect large uncertainties in the early record, although Ansell *et al*. (2006) showed that the winter (DJF) NAO constructed from EMSLP fields shows almost perfect correlation (correlation coefficient = 0.98 for DJF, 1866–2003) with the Jones *et al*. (1997) station‐based NAO index. Furthermore, the correlation between the EMSLP data and the 20CR‐filled Ponta Delgada record is still remarkably high (*r* ^2^ = 0.810). It is unlikely that any of the newly digitized historical Azores data or ISD Azores data were assimilated into the EMSLP and 20CR datasets. This is because the Azores data digitization as part of Projects SIGN and ERA‐CLIM was conducted after production of the 20CR and the online EMSLP project archive (http://www.cru.uea.ac.uk/projects/emulate/) indicates no data for the only contributing Azores station, Angra Do Heroismo, except during 1871–1880 (as such, most of the assimilated observations in the region surrounding the Azores will be maritime‐based, owing to the North Atlantic shipping route). Figure 3 graphically illustrates how the input data in the creation of the Ponta Delgada SLP record vary with time.

**Table 2:** The regression coefficients used to splice together the Ponta Delgada record. The regression relationships were calculated using data up until July 2013. Updating the record with new values (i.e. to present day) simply requires addition of updated SLP data from the Ponta Delgada (Azores) and Reykjavik (Iceland) records from the ISD

Dependent	Predictor	Regression	*r* ^2^
Old PD (1872–1946)	OPD(ISD)0600	HistPD = −1.615 + 1.001 × ISD0600	0.995
Old PD (1872–1946)	OPD(ISD)1200	HistPD = 10.072 + 0.989 × ISD1200	0.968
Old PD (1872–1946)	OPD(ISD)0900	HistPD = −3.120 + 1.002 × ISD0900	0.991
*After all the possible gaps are filled, this becomes the ‘complete’, historical PD time series (1872–1961) [Section * 1.3.1 *]*			
Modern PD (1973–2013)	NPD(ISD)0600	ModernPD = 5.677 + 0.995 × ISD0600	0.989
Modern PD (1973–2013)	NPD(ISD)1200	ModernPD = −0.286 + 1.000 × ISD1200	0.989
Modern PD (1973–2013)	NPD(ISD)0900	ModernPD = −5.950 + 1.005 × ISD0900	0.997
*After all the possible gaps are filled, this becomes the ‘complete’, New PD time series (1973–2013) [Section * 1.3.2 *]*			
New PD (1973–2013)	Lajes	NewPD = 100.048 + 0.902 × Lajes	0.949
New PD (1973–2013)	Santa Maria	NewPD = −48.751 + 1.047 × Santa Maria	0.982
*This extends the New PD record back to August 1944 and fills in almost all daily gaps since then (1944–2013) [Section * 1.3.2 *]*			
New PD (1944–2013)	HistPD (1872–1961)	NewPD = 39.662 + 0.962 × HistPD	0.940
*This results in a long‐term PD record, with only 145 missing days from December 1872 onwards (excluding the long‐term gaps of 1888–1905 and December 1939–1941) (1872–2013) [Section * 1.3.3 *]*			
PD (1872–2013)	20CR (1871–2011)	PD = 47.347 + 0.954 × 20CR	0.931
*This fills in every gap from Jan 1st 1871 to present day (July 2013), creating a continuous, unbroken time series (1871–2013) [Section * 1.3.3 *]*			
PD (1871–2013)	EMSLP (1850–2003)	PD = −52.650 + 1.053 × EMSLP	0.810
*Optionally extends the PD record back to 1850 using EMSLP data (1850–2013) [Section * 1.3.3 *]*			

**Figure 4:**
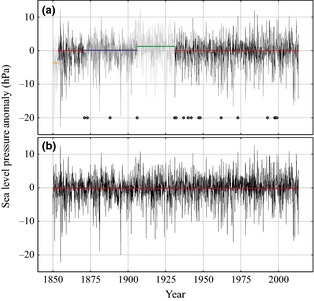
(a) Ponta Delgada monthly SLP time series (as anomalies relative to 1901–2000) before homogenization procedures were applied. Solid lines indicate the difference in means among the three periods (January 1850–March 1853, January 1871–December 1905, and January 1906–December 1930) that underwent homogeneity adjustments. Circles along the −20 hPa axis indicate a change in a dominant data source. (b) Ponta Delgada SLP (anomaly) time series after main homogeneity corrections were applied.

We applied homogeneity tests to the continuous daily and monthly Ponta Delgada time series using the RHTESTSv4 software, which uses the penalized maximum *F* test (Wang, 2008a, 2008b; Wang and Feng, 2013) to detect potential change points. We also used the Standard Normal Homogeneity Test, Buishands Range Test, and the Pettitt Test (Pettitt, 1979; Buishand, 1982; Alexandersson, 1986), finding very similar results (taking significance of break points above the 95% threshold). Typically, when the extra 21 years of data back to 1850 were included; two break points (1853 and 1936) were found and when testing just the 1871–2013 period, break points at 1903 and 1931 were found. As there are switches in data source at 1906 (from 20CR back to the historical Ponta Delgada station) and at 1931 (ISD data, Table 1), we applied slight homogeneity corrections at these times (Figure 4). There is also a change in data source during 1936 (Table 1), but when the correction at 1931 was applied, correcting the ‘shift’ at 1936 was unnecessary. When we extended the Ponta Delgada time series back to 1850 with the EMSLP data, we also adjusted pre‐April 1853 data upwards [a known low bias is present in 1850s EMSLP data (Ansell *et al*., 2006)], as there was a strongly visible ~3 hPa shift. As a further test, we ran the same series of homogeneity tests on monthly series on a month‐by‐month basis to detect if there were any specific ‘time of year’ biases. From this, extra adjustments were made to December and August 1850–1854 (−6.7 and −3.0 hPa, respectively), February 1850–1855 (+10.6 hPa), and October 1857–1860 (+9.5 hPa).

The percentage of the Ponta Delgada record made up from the constituent data sources (up to October 2013) is indicated in Table 3. Nearly, 72% of the 1871–2013 record is directly from a station located in Ponta Delgada, with 12% of the record contributed from the stations on other Azores Islands and 16% from 20CR data.

**Table 3:** The proportion of the Azores record made up by the different data sources (data up to October 2013)

Station	Number of days	Percentage of record (1871–2013)	Percentage of record (1850–2013)
New Ponta Delgada (1973–2013)	13 376	25.64	22.35
Historical Ponta Delgada (1872–1961)	24 129	46.25	40.32
Santa Maria (1944–2013)	1730	3.32	2.89
Lajes (1947–2013)	4753	9.11	7.94
20CR (1871–2011)	8181	15.68	13.67
EMSLP (1850–2003)	7670	0	12.82

### 1.4 New NAO indices

**Figure 5:**
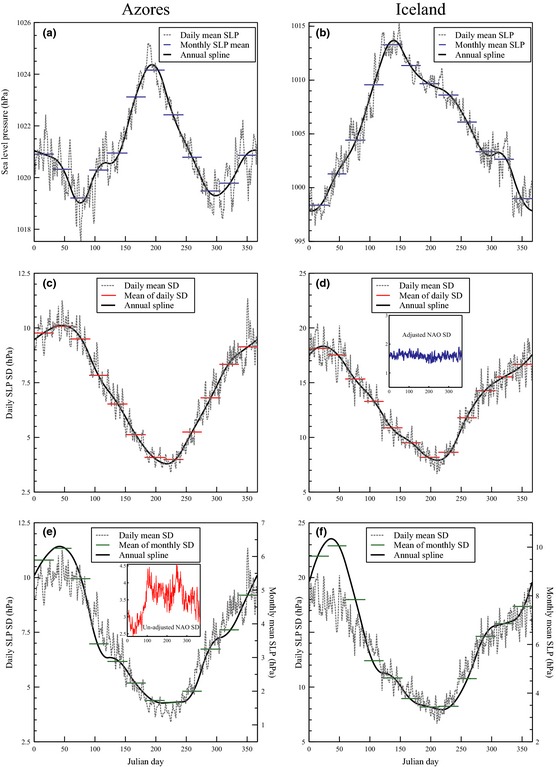
Application of the tension spline method to (a) Azores and (b) Iceland monthly mean (1901–2000 base) SLP pressure, (c) Azores and (d) Iceland monthly SLP SD (where the monthly value is the mean of the daily SLP SD (1901–2000) for each month) and (e) Azores and (f) Iceland monthly SLP SD (where the monthly SD is calculated using monthly SLP data that are aggregated from daily data beforehand). The inset graphs on (d) and (e) display the daily cycle of the SD of the NAO (1850–2013) calculated using the different annual splines. This illustrates the impact of the normalization procedure when calculating the daily NAO. If ‘normal’ monthly SLP SD values (i.e. from Figure (e and f)) are used, then the normalization overly suppresses winter variability (e). If the modified monthly SLP SD values (c and d) are used, then a smooth annual cycle in the NAO index is preserved (d). Note the variable *Y*‐axis for the Azores/Iceland and two NAO inset plots.

The standard method to calculate the monthly NAO is to subtract the normalized monthly value of SLP at Iceland from the Azores. The normalization is done by subtracting the monthly SLP value at each station from its long‐term mean and then dividing by its long‐term standard deviation (SD). Replicating this approach at the daily scale is problematic because even with >160‐year long records, the annual mean cycles of SLP (and SD) as calculated by daily values are irregular (some of these noise features, especially across Iceland, are likely to be real climatological features (Jónsson and Miles, 2001), but such a discussion is beyond the scope of this paper). A smooth annual mean and SD cycle are required for normalization to avoid step changes in the NAO calculation due to day‐to‐day pressure variability. Therefore, we applied a tension spline method (Tveito *et al*., 2001; Henriksen, 2003; Björnsson *et al*., 2007; Björnsson, 2013) where a daily annual cycle (of mean SLP and the SLP SD) was interpolated from monthly values and forced so that the average of the daily values of the curve for each month was equal to the monthly means (Figure 5). Ideally, we would have preferred to use this method on both the monthly mean and SD fields (where the monthly SLP mean and SD were calculated from daily data beforehand) for Iceland and the Azores; however, while this works for the mean SLP, the variability of the monthly SLP SD is problematic (when comparing the annual evolution of the monthly SLP SD with monthly means of the daily SLP SD). Regardless of the base period used (here we used 1901–2000), this issue arises because of the ‘order’ in which the SD is calculated. For example, if we take the SD of January 1st (over the 1901–2000 base period) from Iceland, we get a value of 16.95 hPa. Repeating this across the rest of January and then taking the mean of these 31 values gives a January mean SD of 18.13 hPa. If the raw daily data for January 1901, 1902…2000, are first aggregated to a monthly mean January time series and then the SD of this monthly series (for the same years) is taken, a January mean SD of 9.63 hPa is obtained (the same ‘order’ of calculation has no effect on the mean). This disparity in SD is simply due to the fact that the spread of pressure values for the same day over a large number of years will be greater than the variability of a monthly pressure series. The difference varies disproportionately throughout the year (Figure 5(e)–(f)), and is most evident (least evident) during JFM (MJJASO).

Figure 5(a,b) illustrates the good fit of the tension spline procedure to monthly mean SLP values when compared to the daily annual SLP cycle for the Azores and Iceland. If a normalized NAO is created using the mean SLP splines from Figure 5(a,b) and the ‘monthly’ derived SD splines from Figure 5(e,f), the monthly average of this daily NAO is exactly equal to the monthly NAO (as if it were derived traditionally by converting all daily data to monthly first and applying monthly normalization). This is advantageous; however, due to the (relative) overestimation of the daily SD at each node by the use of the ‘monthly’‐derived SD splines during the first ~90 days of the year, the SD of the daily NAO index is suppressed during the first ~90 days of the year (inset box on Figure 5(e)).

To counter this, we used the daily SD and then calculated the monthly average of the daily SD. We then interpolate back the daily cycle (based on the new monthly SD) using the tension spline methodology, which results in a better fit to the daily annual SD cycle (Figure 5(c,d)). The resulting variability of the NAO index calculated using the SD splines from Figure 5(c,d) is consistent throughout the year (shown by the inset in Figure 5(d)). The only disadvantage of this method is that the monthly average of the daily NAO values is not exactly the same as the monthly NAO values calculated from daily pressure data that are averaged to the monthly scale beforehand (i.e. the ‘traditional’ method). However, from a theoretical and statistical viewpoint, we believe that the ‘adjusted’ daily NAO is a more suitable way to calculate the index, as the annual cycle is adequately preserved.

**Figure 6:**
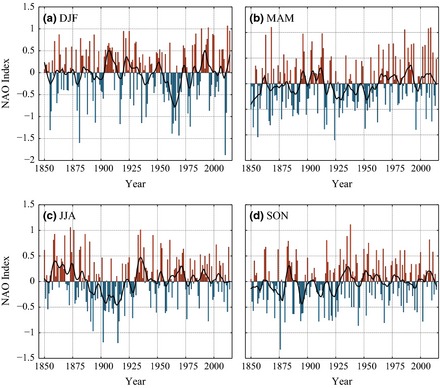
The seasonal North Atlantic Oscillation Index, with a 11‐year loess regression line. The normalization period is 1901–2000.

**Figure 7:**
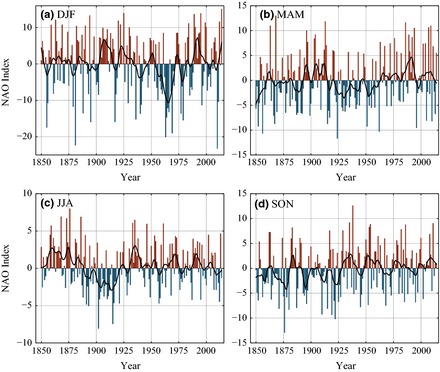
The seasonal natural NAO, with a 11‐year loess regression line.

The difficulty in creating a suitable NAO index at the daily scale highlights the potential hazard of normalization. As such, we also produce a ‘natural’ NAO index (Jónsson and Miles, 2001; Björnsson, 2013), which is simply the daily Azores SLP anomaly minus the Iceland SLP anomaly. This index retains the natural annual pressure cycle so may be more useful for certain climatological applications. The temporal evolution closely matches the normalized NAO (Figures 6 and 7).

To check the robustness of our reconstruction, we compare our daily NAO index (using the monthly average of our daily indices) against five alternative realizations of the NAO (Table 4). Monthly correlations with the Hurrell station index (Hurrell, 1995) and updated Climatic Research Unit (CRU) index (http://www.cru.uea.ac.uk/cru/data/nao/ and http://www.cru.uea.ac.uk/~timo/datapages/naoi.htm), which both use Iceland as the southern node and Lisbon (Hurrell) and the Azores (CRU) as the northern node, remain above 0.90 all year round. A more pronounced seasonal variation is displayed between the Jones *et al*. (1997), Hurrell PC and the CPC indices (http://www.cpc.ncep.noaa.gov/products/precip/CWlink/pna/nao.shtml), due to the use of Gibraltar as the southern station and the PC‐based method, respectively. The difference between the strength of the summer correlation between the two PC‐based indices is most likely a function of how they are calculated – the CPC index uses daily 500 mb height anomalies across 20°–90°N (projected onto a fixed loading pattern) and the Hurrell index uses SLP data across 20°–80°N, 90°W–40°E. As such, the higher correlation values with the Hurrell index are unsurprising, as the spatial domain is restricted to the North Atlantic region and SLP is used as opposed to 500 mb height anomalies (the generally reduced summer correlation across the Gibraltar and PC‐based indices compared to the winter months represents the slight northerly shift in the centres’ of action of the NAO during summer). We examined the temporal difference between our (monthly) NAO and the CRU Azores–Iceland NAO and found no evidence of any significant systematic bias. However, during 1921–1923, our monthly NAO shows much lower values than the CRU version. This arises mainly from the Icelandic node (the early 1920s are a known period of uncertainty in the Icelandic record) and is due to higher SLP values in our version of the Southwest Iceland SLP series compared to the monthly values archived online at CRU.

**Table 4:** The (Pearson) correlation coefficient between the reconstructed NAO presented here (using the monthly average of the daily NAO) with five widely used alternative indices; the updated Hurrell (1995) Lisbon–Iceland station and Principal Component‐based indices, the CRU Azores– and Gibraltar–Iceland indices and the Climate Prediction Centre's NAO index

	Hurrell PC (1899–2013)	Hurrell NAO (1865–2013)	CRU Azores (1865–2010)	CRU Gibraltar (1850–2013)	CPC (1950–2013)
January	0.89	0.99	0.99	0.83	0.91
February	0.91	0.99	0.99	0.86	0.93
March	0.92	0.99	0.99	0.85	0.87
April	0.80	0.98	0.98	0.74	0.64
May	0.83	0.97	0.97	0.67	0.66
June	0.83	0.96	0.96	0.70	0.71
July	0.69	0.90	0.90	0.54	0.35
August	0.66	0.94	0.93	0.52	0.34
September	0.76	0.95	0.95	0.67	0.52
October	0.82	0.97	0.98	0.75	0.72
November	0.82	0.99	0.99	0.78	0.71
December	0.88	0.99	0.99	0.81	0.87

The time periods during which the correlation coefficients are calculated across are indicated. All values are significant (*P* < 0.05).

## 2 Concluding Remarks

The NAO index described here represents our best efforts with currently available data to construct an accurate, continuous, daily NAO index. We anticipate that as data are recovered from global meteorological archives in the future that this index, and similar historical climatic records, can be further refined. While there are numerous versions of the NAO index available, we believe that the importance of the approximately consistent 09:00 UTC observation time gives this index great value. In addition, the index presented here doubles the length of previously available equivalent (daily) datasets and we provide a ‘normal’ (normalised) and ‘natural’ (not normalized) version of the index. Other than the pre‐April 1853 homogeneity adjustment and subsequent minor monthly adjustments, no further attempts were made to remove any outliers in the EMSLP data that contribute to the Azores record during the period 1850–1871. Pre‐December 1872 values in the NAO indices and the periods 1887–1906 and 1940–1941 should be treated with caution as reanalysis output dominates the signal from the southern node, although there is a strong agreement between station‐ and reanalysis‐based SLP for common overlap periods. Extra caution should be applied to the pre‐1871 data, as daily averages are used rather than ~09:00 UTC readings. The ISD data between January 1948 and August 1961, which constitute ~92% of the Ponta Delgada time series during this period, are currently recorded to only the nearest whole hPa, which will have a minor impact on day‐to‐day variability of the Azores SLP time series.

The NAO indices associated with this paper are archived online at the ZENODO repository (http://zenodo.org/record/9979)/DOI:10.5281/zenodo.9979. The uploaded file “NAO.csv” contains date data (across three rows, YYYY, MM, and DD) and the daily value of the normal and natural NAO indices. We anticipate updating the series after each Northern Hemisphere Winter, during April.
